# Long-term patterns of cave-exiting activity of hibernating bats in western North America

**DOI:** 10.1038/s41598-021-87605-0

**Published:** 2021-04-14

**Authors:** Jericho C. Whiting, Bill Doering, Ken Aho, Jason Rich

**Affiliations:** 1 Department of Biology, Brigham Young University-Idaho, 116 Benson Building, Rexburg, ID USA; 2 Wastren Advantage Inc., 120 Technology Drive, Idaho Falls, ID USA; 3 Idaho State University, 921 S. 8th Ave, Mail Stop 8007, Pocatello, ID USA; 4 Air Resources Laboratory Field Research Division, National Oceanic and Atmospheric Administration, 1750 Foote Drive, Idaho Falls, ID USA

**Keywords:** Ecology, Behavioural ecology

## Abstract

Understanding frequency and variation of cave-exiting activity after arousal from torpor of hibernating bats is important for bat ecology and conservation, especially considering white-nose syndrome. In winter from 2011 to 2018, we acoustically monitored, and counted in hibernacula, two species of conservation concern—western small-footed myotis (*Myotis ciliolabrum*) and Townsend’s big-eared bats (*Corynorhinus townsendii*)—in 9 caves located in important habitat for these species in western North America. We investigated if cave-exiting activity differed by species, cave, number of hibernating bats, moon phase, and weather variables. Both species exited hibernacula during all winter months, but most activity occurred in March followed by November. Although we counted almost 15 times more Townsend’s big-eared bats during hibernacula surveys, we documented western small-footed myotis exiting caves 3 times more than Townsend’s big-eared bats. Cave-exiting activity increased with increasing number of hibernating bats, but more so for western small-footed myotis. Both species of bats were active during warm weather and low wind speeds. Western small-footed myotis were more active during colder temperatures, higher wind speeds, and greater change in barometric pressure than Townsend’s big-eared bats. Our results provide a long-term dataset of cave-exiting activity after arousal from torpor during hibernation for these species before the arrival of white-nose syndrome.

## Introduction

Hibernating bats arouse from torpor (i.e., controlled reductions in body temperature and metabolism to conserve energy) and exit caves during winter because of internal cues and environmental changes^1–3^. Some bats arouse in winter and change positions or fly to another hibernaculum^4–6^, often because other bats arouse and fly^7^. Female bats arouse for shorter duration than males^5,8,9^, and younger bats can have shorter torpor bouts than older bats^2^. Some bats arouse from torpor and fly to potentially find food, water^10–12^, or urinate^3^; while others try to mate^2,4^. Arousal from torpor during hibernation may be spontaneous or caused by shifts in temperature and humidity from moving weather fronts^4,6,13^ and changes in barometric pressure^13–15^. Hibernating bats in temperate environments face selective pressures to budget duration, frequency, and timing of torpor arousals to ensure sufficient energy reserves to support survival and subsequent reproduction^7,16–19^.

Humans entering caves for recreation or to conduct research and monitoring can cause bats to arouse from torpor during hibernation^20–22^. Disturbances to bats include lights, noise, vandalism, camping, and caving excursions^20,23,24^. These disturbances can cause bats to arouse from torpor, elevate body temperatures, and use stored energy reserves; thus potentially reducing winter survival^20,23,25^. That reduction in survival can impede population growth, because of low annual reproductive rates of bats^18,24,26^.

A recent threat to bats that causes these mammals to arouse from torpor during hibernation is white-nose syndrome^27–29^. White-nose syndrome is caused by the cold-adapted fungus *Pseudogymnoascus destructans*^30,31^. This fungus invades the integumentary system of infected bats causing tissue damage, increased metabolic rate, and water loss because of excessive wing damange^27,32,33^. Hibernating bats with this disease arouse more often, use more energy because of elevated metabolic rates during torpor, exhibit higher rates of evaporative water loss^32,34,35^, exit caves more often^36,37^, and potentially have reduced reproductive success^38^. Survival of hibernating bats with white-nose syndrome may be influenced by increased arousal and energy expenditure, premature depletion of fat reserves, which can lead to emergence from caves too early and starvation^34,38^. Additionally, infected bats that arouse and fly more can cause conspecifics to arouse, thus negatively influencing fat stores and survival of both^7,39,40^. Primarily a disease occurring in eastern North America, white-nose syndrome is now documented in the western USA^41^.

Bat cave-exiting activity after arousal from torpor during hibernation in western North America is poorly understood^42^, especially in multiple, adjacent caves. We acoustically monitored, and counted bats in, 9 hibernacula that are in an area of important bat habitat^43–45^ in Idaho, USA, during winter from 2011 to 2018. We hypothesised that cave characteristics, number of hibernating bats in each cave, moon phase, and weather variables would influence nightly cave-exiting activity of hibernating Townsend’s big-eared bats (*Corynorhinus townsendii*) and western small-footed myotis (*Myotis ciliolabrum*). Specifically, we predicted that cave-exiting activity would increase for Townsend’s big-eared bats, in large caves, and with more hibernating bats in large clusters^10,40,46,47^. Also, we predicted that bats would be more active during warm, calm nights^3,15^, and that those patterns would hold across all caves. These results provide insight into winter behavior of these species and baseline data of cave-exiting activity after arousal from torpor during hibernation prior to the arrival of white-nose syndrome.

## Methods

### Study area

We monitored cave-exiting activity of bats in 9 hibernacula located in an area of roughly 452 km^2^ on the Snake River Plain in Idaho, USA, on the Idaho National Laboratory Site (43° 36.015 N, 112° 51.441 W). That site was established in the 1940s by the U.S. Atomic Energy Commission as the National Reactor Testing Station, is about 2305 km^2^, and has been closed to public access since that time^48^. Caves in our study area were formed from lava blisters produced by pockets of trapped gas or from tubes of molten flows of basaltic lava that were uncovered when the ceiling collapsed^49,50^. We classified caves as two types: lava blisters or collapsed lava-tubes (Table 1). Lava blisters had small openings (≤ 8 m long x ≤ 6 m wide) in the roof. Conversely, collapsed lava tubes had large openings (≤ 92 m long × 19 m wide) where the roof collapsed forming a crater. Cave ceiling height ranged from about 50 cm to > 10 m. All caves had only one entrance; mean (± *SD*) cave length was 216 m (± 179.2 m, range 25 to 615 m), and mean elevation at cave openings was 1616 m (± 44 m, range = 1551 to 1701 m). The mean distance from a cave to all other caves was 15 km (*SD* = 4.6 km). Our study area was a cold desert consisting mainly of sagebrush (*Artemisia tridentata*)-steppe vegetation^44^. Weather patterns were hot, dry summers and cold winters^48,49^. Most precipitation occurred during winter as snow and during spring as rain or snow^44^. As several of our study caves contain some of the largest hibernating colonies of Townsend’s big-eared bats and western small-footed myotis in western North America^43,50^, we do not provide cave names to protect those resources^25^; however, we assigned letter and number combinations to caves that corresponded with cave letter and number combinations in Whiting et al.^43^. We conducted hibernacula surveys on one day in winter (1 November to 31 March) in 2012, 2013, 2014, 2015, 2017, and 2018^43,44^. Mean date of surveys was February 25. All nine caves were surveyed in a consistent manner each survey. Investigators visually identified and counted bats^43,44^, and all surveys were performed in accordance to established protocols to minimise disturbance of hibernating bats^25,51^. Entering caves to count hibernating bats was approved by the Idaho National Laboratory Site Cave Protection and Access Committee (permit number OS-ESD-16-108). That committee oversees, and grants access into, caves for research on the Idaho National Laboratory Site. Townsend’s big-eared bats and western small-footed myotis comprised > 99% of bats observed during hibernacula surveys in our study^43^.

**Table 1:** Mean (± *SD*) number of Townsend’s big-eared bats (COTO) and western small-footed myotis (MYCI) counted in hibernacula surveys in 9 caves across years we sampled with acoustic detectors, number of files of acoustic recordings for each species, and number of nights (sunset to sunrise) detectors functioned by month from 2011 to 2018 in southeastern Idaho, USA.

Cave^a^	Number counted		Number of files		Number of nights acoustic detectors functioned				
	COTO	MYCI	COTO	MYCI	November	December	January	February	March
C40	383 (41)	15 (6)	2836	4753	69	68	64	74	63
C54	110 (22)	27 (19)	898	6865	58	35	72	71	41
C2	86 (24)	0	107	166	63	89	68	70	78
C19	25 (3)	1 (1)	62	709	58	20	62	42	23
C47	23 (2)	0	159	99	50	36	15	35	26
C46	3 (1)	0	20	315	70	39	45	33	25
C14	2 (2)	0	21	39	81	42	53	30	28
C41	2 (1)	0	34	26	74	23	44	47	36
C36	5	0	23	111	51	24	62	28	19

All caves were collapsed lava-tube caves; except C14, C19, C36, and C41, which were lava blister caves.

^a^Cave letter and numbers correspond with those in Whiting et al.^43^.

### Passive acoustic sampling

We set acoustic detectors (Anabat SDI and SDII; Titley Scientific, Columbia, MO) outside of caves during winter. All detectors were set within a mean of 3 m (*SD* = 2.5 m) of the cave opening or the cave lip. Detectors were powered by external batteries and solar panels^3,10,52^. Each unit was equipped with a protective cover (BatHat) to reduce damage to equipment from rain, snow, and freezing temperatures^53^; eight directional microphones had reflector plates oriented at 45° angle from the center axis of the microphone^3,53,54^, and the directional microphone at one cave (C54) did not have a reflector plate, because of unique cave characteristics. Detectors were programmed to record at least from sunset to sunrise^16,55,56^, and the division ratio was set at eight^54^. We adjusted the sensitivity to exclude ambient noise^57–59^.

We placed microphones about 3 m above the ground and positioned them so the center axis of the zone of reception was approximately 15° above the horizon^15,16,52^. We oriented microphones to maximise detection near cave entrances or craters while trying to avoid recording near-ground noise and echoes^52,53,58^. At collapsed lava-tube caves, we placed detector units near the lip of the crater so that we sampled the area of the crater. At lava blister caves with smaller openings, we set the detector at the cave opening^52^. When triggered by a bat flying outside of hibernacula, detectors created one, ≤ 15 s. call file, labeled with a date and time stamp.

We filtered call files for bat search-phase calls by species using spectrographic analysis software (AnaLookW^10,15,57^; Supplementary Table S1). Past studies have successfully recorded and identified Townsend’s big-eared bats and western small-footed myotis with Anabat detectors^10,54,58,60^. Additionally, one coauthor (Doering who has > 25 years of experience vetting bat calls in the western USA) manually verified species for all files that passed filters. Winter cave-exiting activity after arousal from torpor of bats can be affected when humans enter caves for research and monitoring^22,23^; in our study, on 17 instances, researchers went into caves on one day to collect samples or conduct hibernacula counts. Therefore, we eliminated data for 24 h after each of those events from our analyses^23^.

### Statistical methods

In our analyses, our response variable was the number of files containing at least one search-phase echolocation sequence of ≥ 2 echolocation pulses for each species^15,37,52,53^, each night that the detector functioned. That response variable was an index of bat activity and not abundance^37,52^. Our predictor variables were detector number, year, cave, cave length (m), cave type (lava blister or collapsed lava tube), mean number of hibernating bats counted in each cave, mean cluster size, and mean number of clusters observed during counts in each cave^3,37,61^. We included mean temperature (°C), mean % relative humidity, mean wind speed (m/s) from ½ hour before sunset to ½ hour after sunrise. We also included maximum minus minimum pressure (hPa) over night, accumulated precipitation (rain and melted snow, mm), and moon phase (fraction of moon illuminated at midnight in Mountain Standard Time, http://aa.usno.navy.mil/data/docs/MoonFraction.php)^3,9,16,24,37,62^. Weather data were collected from the closest (within 20 km) National Oceanic and Atmospheric Administration weather station to our study caves every 5 min. from 1/2 hour before sunset to 1/2 hour after sunrise each day. Preliminary analyses indicated that cave length was correlated (*r* > |0.6|) with mean number of Townsend’s big-eared bats and western small-footed myotis counted during surveys. Additionally, three predictor variables—cave length, mean cluster size, and mean number of clusters during surveys—were also confounded with the factor cave, which was required as a random grouping effect in mixed-effect models; therefore, we eliminated those three variables in model building procedures.

We detected bat activity on 19% of nights for Townsend’s big-eared bats and 29% of nights for western small-footed myotis. To account for those data patterns, we used zero-inflated generalised linear mixed models (GLMMs)^63,64^. We considered the error distributions of GLMMs appropriate for count data. Specifically, we created models that incorporated conventional Poisson errors, as well as negative binomial errors with linear and quadratic parameterization to account for potential overdispersion^15,37,65,66^. We used a log link for the GLMM mean function for all three error distributions. We also applied three forms of zero inflation in models: no zero-inflation, constant zero-inflation (zero-inflation as a function of the model intercept), and zero-inflation as a function of temperature. The last approach assumed that bat activity occurred based on a minimum temperature threshold. For fixed effects and quantitative predictions in GLMM models, we tested null hypotheses of no effect using Wald tests^67^. For the random factor cave, we used a likelihood-ratio test for the hypothesis that bat activity did not vary among caves when holding other model terms constant. For all three error distributions and all three forms of zero-inflation, we used backwards stepwise model selection to find optimal approximating (minimum AIC) models^68,69^. We used R statistical environment for all analyses^70^ with packages MASS^71^, asbio^67^, and glmmTMB^72^ to create zero-inflated GLMMs. We set alpha at 0.05 for all analyses.

## Results

**Figure 1:**
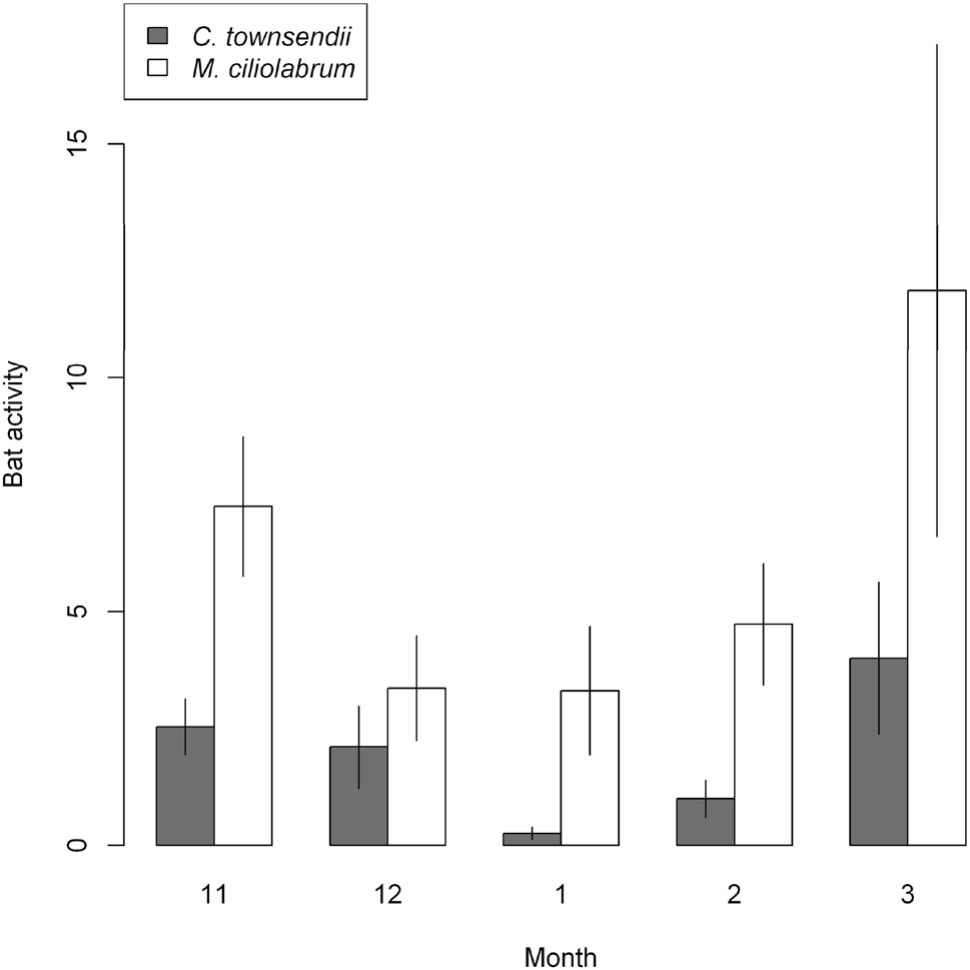
Mean (± 95% CIs) bat activity (files/night) averaged in 9 hibernacula for Townsend’s big-eared bats (*Corynorhinus townsendii*) and western small-footed myotis (*Myotis ciliolabrum*) by month in southeastern Idaho, USA, from 2011 to 2018.

We counted on average almost 15 times more Townsend’s big-eared bats than western small-footed myotis in hibernacula surveys (Table 1). Despite counting more Townsend’s big-eared bats in hibernacula surveys, from 2011 to 2018 at 9 caves, detector units recorded 17,243 files (Townsend’s big-eared bat = 4160 files and western small-footed myotis = 13,083 files; Table 1) during 2204 nights. Mean (± *SD*) number of files recorded per night across caves for Townsend’s big-eared bats was 2 (± 8.3 files, range = 0 to 220 files) and for western small-footed myotis was = 6 (± 24.0 files, range = 0 to 570 files). We recorded Townsend’s big-eared bats and western small-footed myotis in each month of winter, and western small-footed myotis were recorded on average 3 times more than Townsend’s big-eared bats in each month of winter, except in December (Fig. 1).

**Figure 2:**
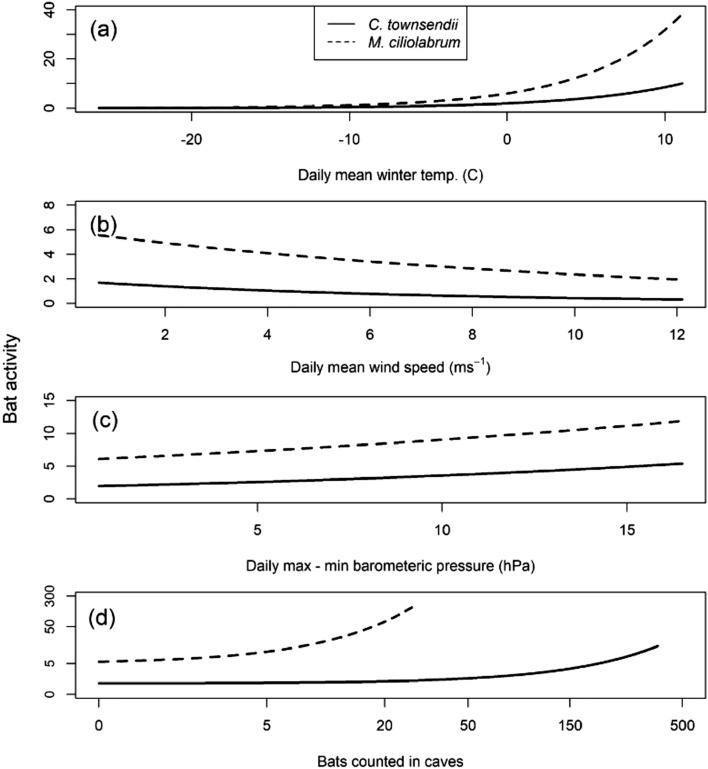
Fitted models while holding predictor variables constant for bat activity (files/night) in 9 hibernacula for Townsend’s big-eared bats (*Corynorhinus townsendii*) and western small-footed myotis (*Myotis ciliolabrum*) by (**a**) temperature, (**b**) wind speed, (**c**) barometric pressure, and (**d**) number of hibernating bats in caves in southeastern Idaho, USA, from 2011 to 2018.

The optimal approximating models (i.e., ∆AIC = 0) for activity of Townsend’s big-eared bats and western small-footed myotis in winter were similar and both contained the same 11 predictor variables (Supplementary Tables S2, S3). For Townsend’s big-eared bats, temperature was the strongest predictor, followed by wind, barometric pressure, and number of hibernating bats in caves (Table 2). For western small-footed myotis, temperature was the strongest predictor, followed by wind, year, number of hibernating bats in caves, and barometric pressure (Table 3). Drop in deviance tests for the random factor cave indicated that activity of Townsend’s big-eared bats (*σ*^2^*REML* = 0.52, *X*^2^ = 67.1, *p* < 0.001) and western small-footed myotis (*σ*^2^*REML* = 0.52, *X*^2^ = 47.7, *p* < 0.001) varied widely across caves. Both species were more active during warm weather, low wind speeds, and greater change in barometric pressure (Fig. 2); western small-footed myotis were more active at colder temperatures, higher wind speeds, greater change in barometric pressure, and when more bats were counted during hibernation than Townsend’s big-eared bats (Fig. 2). At 0 °C, predicted bat activity was 1.9 files/night for Townsend’s big-eared bats and 5.9 files/night for western small-footed myotis (Fig. 2a). At 10 °C, predicted activity increased to 8.5 files/night for Townsend’s big-eared bats and 31.7 files/night for western small-footed myotis (Fig. 2a).

**Table 2:** β coefficients (in log_e_ units) for variables affecting cave-exiting activity in 9 caves during winter for Townsend’s big-eared bats from 2011 to 2018 in southeastern Idaho, USA.

Parameter	Estimate	SE	*z* value	*p* value
(Intercept)	0.59	0.53	1.1	0.27
**# of bats**	**0.0056**	**0.0024**	**2.3**	**0.021**
Year	** − **0.00024	0.043	** − **0.0056	1
Cave type (1)	** − **0.19	0.3	** − **0.65	0.52
**Temperature**	**0.15**	**0.018**	**8.3**	** < 0.0001**
Moon	** − **0.24	0.14	** − **1.8	0.08
Humidity	** − **0.0059	0.0043	** − **1.4	0.17
**Barometric pressure**	**0.064**	**0.018**	**3.6**	** < 0.001**
Precipitation	0.0062	0.057	0.11	0.91
**Wind**	** − 0.15**	**0.027**	** − 5.5**	** < 0.0001**

Significant parameters are bolded.

**Table 3:** β coefficients (in log_e_ units) for variables affecting cave-exiting activity in 9 caves during winter for western small-footed myotis from 2011 to 2018 in southeastern Idaho, USA.

Parameter	Estimate	SE	*z* value	*p* value
(Intercept)	** − **0.15	0.44	** − **0.34	0.73
**# of bats**	**0.12**	**0.029**	**4**	** < 0.0001**
**Year**	**0.12**	**0.029**	**4.2**	** < 0.0001**
Cave type (2)	** − **0.2	0.27	** − **0.73	0.46
**Temperature**	**0.17**	**0.013**	**13**	** < 0.0001**
Moon	0.078	0.11	0.71	0.48
Humidity	0.0057	0.0035	1.6	0.11
**Barometric pressure**	**0.042**	**0.015**	**2.8**	**0.005**
Precipitation	** − **0.021	0.044	** − **0.47	0.64
**Wind**	** − 0.092**	**0.022**	** − 4.3**	** < 0.0001**

Significant parameters are bolded.

## Discussion

Biologists need to understand species-specific differences in bat winter ecology that can influence mortality risk for hibernating bats, especially in western North America^3,42^. We predicted that cave-exiting behavior after arousal from torpor would increase for the larger sized Townsend’s big-eared bats^10^. Although we counted almost 15 times more of that species than western small-footed myotis during hibernacula surveys, because small-footed myotis are more difficult to observe when hibernating^73,74^, we documented western small-footed myotis exiting caves after arousal from torpor during hibernation on average 3 times more than Townsend’s big-eared bats. Different arousal and flying patterns during winter have been documented for other species hibernating together^3,75,76^, and generally it takes more energy for larger bats to arouse and fly than smaller bats^20,77^. Also, difficulty exists when comparing winter activity of bat species using acoustic recordings, because of species-specific differences in intensity of echolocation calls and atmospheric attenuation^57,61,78^, especially for Townsend’s big-eared bats as their calls are lower intensity compared with calls of western small-footed myotis^52,60^. Such differences need to be considered when comparing acoustic activity between species. Nonetheless, understanding frequency and variation of bat winter activity levels across species is important for bat ecology and conservation^10,76,79^, especially in light of white-nose syndrome^9,80^. General conditions of humidity and temperature exist for growth of *Pseudogymnoascus destructans* in the western USA^36,50^, and Townsend’s big-eared bats can carry this fungus^81^. Our results provide long-term baseline data of cave-exiting activity prior to the arrival white-nose syndrome in Idaho, which can be used to compare with changes in bat activity after this fungus arrives as has been done in the eastern USA^80,82,83^.

One of the main differences in winter cave-exiting activity between the two species we studied could have been due to differences in body size and evaporative water loss. Mass of adult Townsend’s big-eared bats ranges from 5 to 13 g, and females are heavier than males in autumn and winter^4,6^. For western small-footed myotis, mass of adult animals is about 4.5 g^84^. Smaller bats may arouse more from torpor during hibernation to drink water. Indeed, a laboratory study indicated that bats must drink every 9–12 days during hibernation^77^, and studies conducted in the field have provided evidence of bat arousing from torpor in winter to drink water^10^. Also, bats that arouse more during hibernation have higher rates of total evaporative water loss^12^, and evaporative water loss may also be driven by humidity levels in caves^12^.

Little is known about bat cave-exiting activity after arousal from torpor among multiple hibernacula with differing numbers of bats. Our prediction that cave-exiting behavior would increase in large caves with more hibernating bats was upheld. Indeed, we documented a positive trend in bat activity by both species with increasing number of hibernating conspecifics in caves, but more so for western small-footed myotis. Large numbers of bats and groups of conspecifics can cause other bats to arouse and fly in winter^40,75^, which may have occurred in our study area. We hypothesise that when white-nose syndrome arrives in Idaho, infected bats in caves with more hibernating individuals will cause conspecifics to arouse more, thus negatively impacting survival of both^7,39,40^. Our data also indicated that cave-exiting behavior varied widely across the 9 caves for western small-footed myotis and Townsend’s big-eared bats. Observed differences in cave-exiting behavior highlights the importance of quantifying bat activity at caves with differing number of hibernating bats to understand the influence of habitat and environmental variables, as well as disease, on local bat populations^52^.

We documented highest levels of bat activity during November and March, and lowest levels during the coldest winter months of December, January, and February, which has been documented in other studies in temperate, northern environments^10,16,52^. Also, less variation was evident in activity of Townsend’s big-eared bats both within and among months. Bats often fly and forage at the beginning and end of hibernation season on warm, calm nights^77^. During the coldest months of winter, however, bats go farther into caves^6,24^. Timing arousal events to coincide with high ambient temperatures reduces the total energy expense of reaching euthermia^3^. Indeed, relying on increased ambient temperature to elevate body temperature (passive rewarming) can save 20% of the energetic cost of arousal^85^. Species that hibernate assess environmental conditions at or near the entrances of hibernacula to more accurately time emergence. Also, changes in barometric pressure could signal favorable conditions for bat emergence, especially for individuals that roost deep in caves^13,86^. The more frequently that this assessment is done, the more accurately that emergence can be timed^47^.

We predicted that bats would be more active during warm, calm nights. In our study, temperature, wind, and change in barometric pressure were strong predictors of bat activity for both species; however, western small-footed myotis were more active at colder temperatures, higher wind speeds, and higher change in barometric pressure than Townsend’s big-eared bats. Our result differed from another study that documented the larger sized big brown bat (*Eptesicus fuscus*) as more active at higher temperatures than *Myotis* spp.^15^. Temperature and wind speed were predictors of bat activity in other studies^13,15,62^, and bats responded to weather patterns^62^ and changes in barometric pressure^13,15^. Bat calls have been recorded at temperatures below 0 °C^15^, but most activity occurred on nights when the temperature at sunset exceeded 0 °C^10,62^, and the probability of activity increases as temperature increases^62^, similar to what we documented. Warmer ambient temperatures can also increase frequency of arousals within hibernacula; we were not able to relate the frequency of arousals of bats within hibernacula to activity outside of those features, because we did not acoustically monitor bats inside hibernacula. Future studies need to test how cave-exiting activity by bats relates to frequency of arousals and bats flying in hibernacula.

Much interest exists in developing long-term acoustic monitoring of bats^56,82,87^, and deploying several stationary detectors is valuable for understanding bat activity at a landscape scale^61^. With the arrival of white-nose syndrome in western North America^41^, it is important to understand cave-exiting behavior of bats after arousal from torpor^9,75^. Furthermore, comparisons among species need to be conducted at large geographic scales to determine differences in winter activity strategies^88^. We acoustically monitored, and counted bats in, 9 hibernacula that were in an area of important habitat during winter. We recorded western small-footed myotis exiting caves 3 times more than Townsend’s big-eared bats, and cave-exiting behavior increased similarly with increasing number of hibernating bats for these species. Temperature, wind speed, and change in barometric pressure were strong predictors of bat activity for both species. Our results provide insight into cave-exiting activity after arousal from torpor of these species and provide a long-term baseline dataset of that activity prior to the arrival of white-nose syndrome. Such data can help biologists when quantifying the potential impact of white-nose syndrome on these species.

## Supplementary Information

Supplementary Information.

## Data Availability

The datasets generated during and/or analysed during the current study are available from the corresponding author.
