# Supplementary material for: Long-term patterns of cave-exiting activity of hibernating bats in western North America
Source: Sci Rep. 2021 Apr 14;11:8175. doi: 10.1038/s41598-021-87605-0 (PMC8046793; doi:10.1038/s41598-021-87605-0)
Supplement: Supplementary file 1 — Supplementary Information. [file 41598_2021_87605_MOESM1_ESM.docx]

**Long-term patterns of cave-exiting activity of hibernating bats in western North America**

Jericho C. Whiting^1*^, Bill Doering^2^, Ken Aho^3^, & Jason Rich^4^

**Supplementary information.** Further exploration of bat call file analyses and model selection.

| Filters | Min. # of pulses |  |  |  |  |  |  |
| --- | --- | --- | --- | --- | --- | --- | --- |
|  |  | F_c_ | S_c_ | F_min_ | F_max_ | Sweep | Duration (ms) |
| Townsend’s big-eared | 2 | 27 to 36 | 60 to 650 | 25 to 36 | 31 to 47 | 4.5 to 35 | 0.6 to 10 |
| 40 kHz myotis | 3 | 38 to 56 | 26 to 600 | - | 45 to 120 | 4.5 to 70 | 0.6 to 10 |

**Table S1**. Parameters of bat calls used to create filters for our analyses of bat cave-exiting activity from 2011 to 2018 in southeastern Idaho, USA. Parameters included minimum number of pulses in 15 sec. (min. # of pulses), and range of values for characteristic frequency (F_c_), slope of call body (S_c_), minimum frequency of calls (F_min_), maximum frequency of calls (F_max_), sweep, and duration of calls ^1-9^.

**Table S2.** Modeling results for cave-exiting activity of Townsend’s big-eared bats during hibernation from 2011 to 2018 in southeastern Idaho, USA. A plus sign indicates the presence of a predictor variable in the model. Rank = model rank, Wind = mean wind speed (m/sec.), Prec. = accumulated precipitation (rain and melted snow, mm), Hum. = mean % relative humidity, Barom. = maximum minus minimum pressure (hPa), Moon = moon phase (fraction of moon illuminated at midnight in Mountain Standard Time), Year = study year, Type = cave type, # of bats = mean number of bats counted during surveys, Temp. = mean temperature (°C), Random effects = random effects described as they would be specified in widely-used functions for random and mixed effect models in R (e.g., lmer, glmer, glmmTMB), Family = family of statistical models (NB1 = negative binomial distribution with linear parameterization, NB2 = negative binomial distribution with quadratic parameterization, and generalised Poisson distribution models), Zero-inflation = zeroes indicate that no form of zero inflation was specified, “∼1 ” indicates that constant zero-inflation (an intercept-only model for the zero-inflation) was used, and “∼Temp” indicates that zero inflation was modeled as a function of temperature, AIC = Akaike’s information criterion, and ∆ AIC = delta AIC.

| Rank | Wind | Prec. | Hum. | Barom. | Moon | Year | Type | # of bats | Temp. | Random effect(s) | Family (link) | Zero-inflation | AIC | ∆ AIC |
| --- | --- | --- | --- | --- | --- | --- | --- | --- | --- | --- | --- | --- | --- | --- |
| 1 | + | + | + | + | + | + | + | + | + | 1\|Cave, 1\|Detector | NB1 (log) | ~Temp | 3457.8 | 0.0 |
| 2 | + | + | + | + | + | + | + | + | + | 1\|Cave, 1\|Detector | NB2 (log) | ~Temp | 3480.8 | 22.9 |
| 3 | + | + | + | + | + | + | + | + | + | 1\|Cave, 1\|Detector | NB1 (log) | ~0 | 3511.8 | 53.9 |
| 4 | + | + | + | + | + | + | + | + | + | 1\|Cave, 1\|Detector | NB1 (log) | ~1 | 3512.7 | 54.9 |
| 5 | + | + | + | + | + | + | + | + | + | 1\|Cave, 1\|Detector | NB2 (log) | ~0 | 3577.4 | 119.6 |
| 6 | + | + | + | + | + | + | + | + | + | 1\|Cave, 1\|Detector | NB2 (log) | ~1 | 3579.4 | 121.6 |
| 7 | - | - | + | + | + | + | + | + | + | 1\|Cave | NB1 (log) | ~Temp | 3671.0 | 213.2 |
| 8 | - | + | + | + | + | + | - | + | + | 1\|Cave | NB1 (log) | ~Temp | 3671.3 | 213.5 |
| 9 | - | + | - | + | + | + | + | + | + | 1\|Cave | NB1 (log) | ~Temp | 3672.3 | 214.5 |
| 10 | + | + | + | + | + | + | + | + | + | 1\|Cave | NB1 (log) | ~Temp | 3672.9 | 215.1 |
| 11 | - | + | + | + | - | + | + | + | + | 1\|Cave | NB1 (log) | ~Temp | 3676.8 | 219.0 |
| 12 | - | + | + | + | + | + | + | - | + | 1\|Cave | NB1 (log) | ~Temp | 3677.6 | 219.8 |
| 13 | - | + | + | - | + | + | + | + | + | 1\|Cave | NB1 (log) | ~Temp | 3680.6 | 222.8 |
| 14 | - | + | + | + | + | - | + | + | + | 1\|Cave | NB1 (log) | ~Temp | 3704.9 | 247.0 |
| 15 | - | - | + | + | + | + | + | + | + | 1\|Cave | NB2 (log) | ~Temp | 3723.4 | 265.6 |
| 16 | - | + | + | + | + | - | + | + | + | 1\|Cave | NB2 (log) | ~Temp | 3723.7 | 265.8 |
| 17 | - | + | + | + | + | + | - | + | + | 1\|Cave | NB2 (log) | ~Temp | 3724.2 | 266.4 |
| 18 | + | + | + | + | + | + | + | + | + | 1\|Cave | NB2 (log) | ~Temp | 3725.1 | 267.3 |
| 19 | - | - | + | + | + | + | + | + | + | 1\|Cave | NB1 (log) | ~0 | 3726.6 | 268.7 |
| 20 | - | + | + | + | + | + | - | + | + | 1\|Cave | NB1 (log) | ~0 | 3726.7 | 268.9 |
| 21 | + | + | + | + | + | + | + | + | + | 1\|Cave | NB1 (log) | ~0 | 3728.2 | 270.4 |
| 22 | - | + | + | - | + | + | + | + | + | 1\|Cave | NB2 (log) | ~Temp | 3728.5 | 270.6 |
| 23 | - | - | + | + | + | + | + | + | + | 1\|Cave | NB1 (log) | ~1 | 3728.5 | 270.7 |
| 24 | - | + | + | + | + | + | - | + | + | 1\|Cave | NB1 (log) | ~1 | 3728.7 | 270.9 |
| 25 | - | + | - | + | + | + | + | + | + | 1\|Cave | NB2 (log) | ~Temp | 3729.2 | 271.4 |
| 26 | + | + | + | + | + | + | + | + | + | 1\|Cave | NB1 (log) | ~1 | 3730.2 | 272.4 |
| 27 | - | + | + | + | + | + | + | - | + | 1\|Cave | NB2 (log) | ~Temp | 3730.5 | 272.7 |
| 28 | - | + | + | + | - | + | + | + | + | 1\|Cave | NB1 (log) | ~0 | 3731.2 | 273.4 |
| 29 | - | + | + | + | - | + | + | + | + | 1\|Cave | NB2 (log) | ~Temp | 3731.6 | 273.8 |
| 30 | - | + | - | + | + | + | + | + | + | 1\|Cave | NB1 (log) | ~0 | 3731.7 | 273.9 |
| 31 | - | + | + | + | + | + | + | - | + | 1\|Cave | NB1 (log) | ~0 | 3732.6 | 274.8 |
| 32 | - | + | + | + | - | + | + | + | + | 1\|Cave | NB1 (log) | ~1 | 3733.2 | 275.4 |
| 33 | - | + | - | + | + | + | + | + | + | 1\|Cave | NB1 (log) | ~1 | 3733.4 | 275.5 |
| 34 | - | + | + | + | + | + | + | - | + | 1\|Cave | NB1 (log) | ~1 | 3734.6 | 276.8 |
| 35 | - | + | + | - | + | + | + | + | + | 1\|Cave | NB1 (log) | ~0 | 3740.9 | 283.1 |
| 36 | - | + | + | - | + | + | + | + | + | 1\|Cave | NB1 (log) | ~1 | 3742.9 | 285.1 |
| 37 | - | + | + | + | + | + | + | + | + | 1\|Cave | NB1 (log) | ~Temp | 3745.3 | 287.4 |
| 38 | - | + | + | + | + | - | + | + | + | 1\|Cave | NB1 (log) | ~0 | 3755.4 | 297.6 |
| 39 | - | + | + | + | + | - | + | + | + | 1\|Cave | NB1 (log) | ~1 | 3757.1 | 299.3 |
| 40 | - | + | + | + | + | + | + | + | - | 1\|Cave | NB1 (log) | ~Temp | 3760.0 | 302.1 |
| 41 | - | + | + | + | + | + | + | + | - | 1\|Cave | NB2 (log) | ~Temp | 3791.9 | 334.0 |
| 42 | - | + | + | + | + | + | + | + | + | 1\|Cave | NB2 (log) | ~Temp | 3797.2 | 339.4 |
| 43 | - | + | + | + | + | + | + | + | + | 1\|Cave | NB1 (log) | ~0 | 3811.2 | 353.4 |
| 44 | - | + | + | + | + | + | + | + | + | 1\|Cave | NB1 (log) | ~1 | 3813.2 | 355.4 |
| 45 | - | - | + | + | + | + | + | + | + | 1\|Cave | NB2 (log) | ~0 | 3828.0 | 370.2 |
| 46 | - | + | - | + | + | + | + | + | + | 1\|Cave | NB2 (log) | ~0 | 3828.3 | 370.4 |
| 47 | - | + | + | + | + | + | - | + | + | 1\|Cave | NB2 (log) | ~0 | 3828.7 | 370.9 |
| 48 | - | + | + | + | + | - | + | + | + | 1\|Cave | NB2 (log) | ~0 | 3829.7 | 371.9 |
| 49 | + | + | + | + | + | + | + | + | + | 1\|Cave | NB2 (log) | ~0 | 3829.8 | 372.0 |
| 50 | - | - | + | + | + | + | + | + | + | 1\|Cave | NB2 (log) | ~1 | 3830.0 | 372.2 |
| 51 | - | + | - | + | + | + | + | + | + | 1\|Cave | NB2 (log) | ~1 | 3830.3 | 372.4 |
| 52 | - | + | + | + | + | + | - | + | + | 1\|Cave | NB2 (log) | ~1 | 3830.7 | 372.9 |
| 53 | - | + | + | + | + | - | + | + | + | 1\|Cave | NB2 (log) | ~1 | 3831.7 | 373.9 |
| 54 | + | + | + | + | + | + | + | + | + | 1\|Cave | NB2 (log) | ~1 | 3831.8 | 374.0 |
| 55 | - | + | + | + | + | + | + | - | + | 1\|Cave | NB2 (log) | ~0 | 3834.4 | 376.6 |
| 56 | - | + | + | + | + | + | + | - | + | 1\|Cave | NB2 (log) | ~1 | 3836.4 | 378.6 |
| 57 | - | + | + | + | - | + | + | + | + | 1\|Cave | NB2 (log) | ~0 | 3839.0 | 381.2 |
| 58 | - | + | + | + | - | + | + | + | + | 1\|Cave | NB2 (log) | ~1 | 3841.0 | 383.2 |
| 59 | - | + | + | - | + | + | + | + | + | 1\|Cave | NB2 (log) | ~0 | 3842.5 | 384.6 |
| 60 | - | + | + | - | + | + | + | + | + | 1\|Cave | NB2 (log) | ~1 | 3844.5 | 386.6 |
| 61 | - | + | + | + | + | + | + | + | + | 1\|Cave | NB2 (log) | ~0 | 3914.3 | 456.5 |
| 62 | - | + | + | + | + | + | + | + | + | 1\|Cave | NB2 (log) | ~1 | 3916.3 | 458.5 |
| 63 | - | + | + | + | + | + | + | + | - | 1\|Cave | NB1 (log) | ~1 | 4209.5 | 751.7 |
| 64 | - | + | + | + | + | + | + | + | - | 1\|Cave | NB2 (log) | ~0 | 4233.0 | 775.2 |
| 65 | - | + | + | + | + | + | + | + | - | 1\|Cave | NB2 (log) | ~1 | 4233.4 | 775.5 |
| 66 | - | + | + | + | + | + | + | + | - | 1\|Cave | NB1 (log) | ~0 | 4237.0 | 779.1 |
| 67 | + | + | + | + | + | + | + | + | + | 1\|Cave, 1\|Detector | POI (log) | ~Temp | 5571.2 | 2113.3 |
| 68 | + | + | + | + | + | + | + | + | + | 1\|Cave, 1\|Detector | POI (log) | ~1 | 5848.5 | 2390.6 |
| 69 | + | + | + | + | + | + | + | + | + | 1\|Cave | POI (log) | ~Temp | 6364.8 | 2907.0 |
| 70 | - | + | + | + | + | + | - | + | + | 1\|Cave | POI (log) | ~Temp | 6365.0 | 2907.2 |
| 71 | - | + | + | + | + | + | + | - | + | 1\|Cave | POI (log) | ~Temp | 6368.4 | 2910.6 |
| 72 | - | - | + | + | + | + | + | + | + | 1\|Cave | POI (log) | ~Temp | 6376.5 | 2918.7 |
| 73 | - | + | + | - | + | + | + | + | + | 1\|Cave | POI (log) | ~Temp | 6388.2 | 2930.4 |
| 74 | - | + | + | + | - | + | + | + | + | 1\|Cave | POI (log) | ~Temp | 6404.2 | 2946.3 |
| 75 | - | + | + | + | + | + | + | + | + | 1\|Cave | POI (log) | ~Temp | 6431.0 | 2973.1 |
| 76 | - | + | - | + | + | + | + | + | + | 1\|Cave | POI (log) | ~Temp | 6452.4 | 2994.6 |
| 77 | - | + | + | + | + | - | + | + | + | 1\|Cave | POI (log) | ~Temp | 6469.4 | 3011.6 |
| 78 | - | + | + | + | + | + | + | + | - | 1\|Cave | POI (log) | ~Temp | 6626.7 | 3168.9 |
| 79 | + | + | + | + | + | + | + | + | + | 1\|Cave | POI (log) | ~1 | 6668.3 | 3210.4 |
| 80 | - | + | + | + | + | + | - | + | + | 1\|Cave | POI (log) | ~1 | 6669.1 | 3211.2 |
| 81 | - | + | + | + | + | + | + | - | + | 1\|Cave | POI (log) | ~1 | 6673.3 | 3215.4 |
| 82 | - | - | + | + | + | + | + | + | + | 1\|Cave | POI (log) | ~1 | 6678.1 | 3220.2 |
| 83 | - | + | + | - | + | + | + | + | + | 1\|Cave | POI (log) | ~1 | 6701.0 | 3243.1 |
| 84 | - | + | + | + | - | + | + | + | + | 1\|Cave | POI (log) | ~1 | 6708.9 | 3251.1 |
| 85 | - | + | - | + | + | + | + | + | + | 1\|Cave | POI (log) | ~1 | 6726.5 | 3268.6 |
| 86 | - | + | + | + | + | + | + | + | + | 1\|Cave | POI (log) | ~1 | 6745.7 | 3287.9 |
| 87 | - | + | + | + | + | - | + | + | + | 1\|Cave | POI (log) | ~1 | 6777.3 | 3319.5 |
| 88 | - | + | + | + | + | + | + | + | - | 1\|Cave | POI (log) | ~1 | 7024.5 | 3566.7 |
| 89 | + | + | + | + | + | + | + | + | + | 1\|Cave, 1\|Detector | POI (log) | ~0 | 7223.1 | 3765.3 |
| 90 | - | + | - | + | + | + | + | + | + | 1\|Cave | POI (log) | ~0 | 8510.9 | 5053.1 |
| 91 | - | + | + | + | + | + | - | + | + | 1\|Cave | POI (log) | ~0 | 8511.2 | 5053.4 |
| 92 | + | + | + | + | + | + | + | + | + | 1\|Cave | POI (log) | ~0 | 8511.8 | 5054.0 |
| 93 | - | - | + | + | + | + | + | + | + | 1\|Cave | POI (log) | ~0 | 8512.1 | 5054.3 |
| 94 | - | + | + | + | + | + | + | - | + | 1\|Cave | POI (log) | ~0 | 8518.0 | 5060.1 |
| 95 | - | + | + | + | - | + | + | + | + | 1\|Cave | POI (log) | ~0 | 8585.4 | 5127.6 |
| 96 | - | + | + | - | + | + | + | + | + | 1\|Cave | POI (log) | ~0 | 8629.0 | 5171.2 |
| 97 | - | + | + | + | + | + | + | + | + | 1\|Cave | POI (log) | ~0 | 8704.1 | 5246.3 |
| 98 | - | + | + | + | + | - | + | + | + | 1\|Cave | POI (log) | ~0 | 8746.4 | 5288.6 |
| 99 | - | + | + | + | + | + | + | + | - | 1\|Cave | POI (log) | ~0 | 11469.3 | 8011.5 |

**Table S3.** Modeling results for cave-exiting activity of western small-footed myotis during hibernation from 2011 to 2018 in southeastern Idaho, USA. A plus sign indicates the presence of a predictor variable in the model. Rank = model rank, Wind = mean wind speed (m/sec.), Prec. = accumulated precipitation (rain and melted snow, mm), Hum. = mean % relative humidity, Barom. = maximum minus minimum pressure (hPa), Moon = moon phase (fraction of moon illuminated at midnight in Mountain Standard Time), Year = study year, Type = cave type, # of bats = mean number of bats counted during surveys, Temp. = mean temperature (°C), Random effects = random effects described as they would be specified in widely-used functions for random and mixed effect models in R (e.g., lmer, glmer, glmmTMB), Family = family of statistical models (NB1 = negative binomial distribution with linear parameterization, NB2 = negative binomial distribution with quadratic parameterization, and generalised Poisson distribution models), Zero-inflation = zeroes indicate that no form of zero inflation was specified, “∼1 ” indicates that constant zero-inflation (an intercept-only model for the zero-inflation) was used, and “∼Temp” indicates that zero inflation was modeled as a function of temperature, AIC = Akaike’s information criterion, and ∆ AIC = delta AIC.

| Rank | Wind | Prec. | Hum. | Barom. | Moon | Year | Type | # of bats | Temp. | Random effect(s) | Family (link) | Zero-inflation | AIC | ∆ AIC |
| --- | --- | --- | --- | --- | --- | --- | --- | --- | --- | --- | --- | --- | --- | --- |
| 1 | + | + | + | + | + | + | + | + | + | 1\|Cave, 1\|Detector | NB1 (log) | ~Temp | 5687.8 | 0.0 |
| 2 | + | + | + | + | + | + | + | + | + | 1\|Cave, 1\|Detector | NB1 (log) | ~1 | 5720.2 | 32.4 |
| 3 | + | + | + | + | + | + | + | + | + | 1\|Cave, 1\|Detector | NB1 (log) | ~0 | 5721.3 | 33.5 |
| 4 | + | + | + | + | + | + | + | + | + | 1\|Cave, 1\|Detector | NB2 (log) | ~Temp | 5748.4 | 60.6 |
| 5 | + | + | + | + | + | + | + | + | + | 1\|Cave, 1\|Detector | NB2 (log) | ~0 | 5839.9 | 152.1 |
| 6 | + | + | + | + | + | + | + | + | + | 1\|Cave, 1\|Detector | NB2 (log) | ~1 | 5841.9 | 154.1 |
| 7 | - | - | + | + | + | + | + | + | + | 1\|Cave | NB1 (log) | ~Temp | 5994.5 | 306.7 |
| 8 | - | + | + | + | - | + | + | + | + | 1\|Cave | NB1 (log) | ~Temp | 5994.5 | 306.8 |
| 9 | - | + | + | + | + | + | - | + | + | 1\|Cave | NB1 (log) | ~Temp | 5994.8 | 307.0 |
| 10 | + | + | + | + | + | + | + | + | + | 1\|Cave | NB1 (log) | ~Temp | 5996.3 | 308.5 |
| 11 | - | + | - | + | + | + | + | + | + | 1\|Cave | NB1 (log) | ~Temp | 5998.2 | 310.4 |
| 12 | - | + | + | - | + | + | + | + | + | 1\|Cave | NB1 (log) | ~Temp | 5998.8 | 311.0 |
| 13 | - | + | + | + | + | + | + | - | + | 1\|Cave | NB1 (log) | ~Temp | 6002.0 | 314.2 |
| 14 | - | + | + | + | + | - | + | + | + | 1\|Cave | NB1 (log) | ~Temp | 6011.7 | 323.9 |
| 15 | - | + | + | + | - | + | + | + | + | 1\|Cave | NB1 (log) | ~0 | 6029.5 | 341.8 |
| 16 | - | + | + | + | + | + | - | + | + | 1\|Cave | NB1 (log) | ~0 | 6029.8 | 342.0 |
| 17 | - | - | + | + | + | + | + | + | + | 1\|Cave | NB1 (log) | ~0 | 6030.3 | 342.5 |
| 18 | - | + | + | + | - | + | + | + | + | 1\|Cave | NB1 (log) | ~1 | 6031.0 | 343.2 |
| 19 | + | + | + | + | + | + | + | + | + | 1\|Cave | NB1 (log) | ~0 | 6031.3 | 343.5 |
| 20 | - | + | + | + | + | + | - | + | + | 1\|Cave | NB1 (log) | ~1 | 6031.3 | 343.5 |
| 21 | - | - | + | + | + | + | + | + | + | 1\|Cave | NB1 (log) | ~1 | 6031.9 | 344.1 |
| 22 | + | + | + | + | + | + | + | + | + | 1\|Cave | NB1 (log) | ~1 | 6032.7 | 345.0 |
| 23 | - | + | + | + | + | + | + | - | + | 1\|Cave | NB1 (log) | ~0 | 6036.8 | 349.1 |
| 24 | - | + | + | - | + | + | + | + | + | 1\|Cave | NB1 (log) | ~0 | 6037.8 | 350.0 |
| 25 | - | + | + | + | + | + | + | - | + | 1\|Cave | NB1 (log) | ~1 | 6038.3 | 350.5 |
| 26 | - | + | + | - | + | + | + | + | + | 1\|Cave | NB1 (log) | ~1 | 6039.4 | 351.6 |
| 27 | - | + | - | + | + | + | + | + | + | 1\|Cave | NB1 (log) | ~0 | 6045.6 | 357.8 |
| 28 | - | + | - | + | + | + | + | + | + | 1\|Cave | NB1 (log) | ~1 | 6046.3 | 358.5 |
| 29 | - | + | + | + | + | - | + | + | + | 1\|Cave | NB1 (log) | ~0 | 6046.5 | 358.7 |
| 30 | - | + | + | + | + | - | + | + | + | 1\|Cave | NB1 (log) | ~1 | 6047.7 | 359.9 |
| 31 | - | + | + | + | - | + | + | + | + | 1\|Cave | NB2 (log) | ~Temp | 6058.4 | 370.6 |
| 32 | - | + | + | + | + | + | - | + | + | 1\|Cave | NB2 (log) | ~Temp | 6059.1 | 371.3 |
| 33 | - | + | + | - | + | + | + | + | + | 1\|Cave | NB2 (log) | ~Temp | 6059.5 | 371.7 |
| 34 | + | + | + | + | + | + | + | + | + | 1\|Cave | NB2 (log) | ~Temp | 6060.2 | 372.4 |
| 35 | - | + | - | + | + | + | + | + | + | 1\|Cave | NB2 (log) | ~Temp | 6060.7 | 373.0 |
| 36 | - | - | + | + | + | + | + | + | + | 1\|Cave | NB2 (log) | ~Temp | 6062.5 | 374.7 |
| 37 | - | + | + | + | + | - | + | + | + | 1\|Cave | NB2 (log) | ~Temp | 6062.9 | 375.1 |
| 38 | - | + | + | + | + | + | + | - | + | 1\|Cave | NB2 (log) | ~Temp | 6066.3 | 378.5 |
| 39 | - | + | + | + | + | + | + | + | + | 1\|Cave | NB1 (log) | ~Temp | 6123.6 | 435.8 |
| 40 | - | + | + | + | - | + | + | + | + | 1\|Cave | NB2 (log) | ~0 | 6158.8 | 471.0 |
| 41 | - | + | + | + | + | + | - | + | + | 1\|Cave | NB2 (log) | ~0 | 6159.9 | 472.1 |
| 42 | + | + | + | + | + | + | + | + | + | 1\|Cave | NB2 (log) | ~0 | 6160.8 | 473.0 |
| 43 | - | + | + | + | - | + | + | + | + | 1\|Cave | NB2 (log) | ~1 | 6160.8 | 473.0 |
| 44 | - | + | + | + | + | - | + | + | + | 1\|Cave | NB2 (log) | ~0 | 6161.3 | 473.6 |
| 45 | - | + | + | + | + | + | - | + | + | 1\|Cave | NB2 (log) | ~1 | 6161.9 | 474.1 |
| 46 | - | + | + | - | + | + | + | + | + | 1\|Cave | NB2 (log) | ~0 | 6162.5 | 474.7 |
| 47 | + | + | + | + | + | + | + | + | + | 1\|Cave | NB2 (log) | ~1 | 6162.8 | 475.0 |
| 48 | - | + | + | + | + | - | + | + | + | 1\|Cave | NB2 (log) | ~1 | 6163.3 | 475.6 |
| 49 | - | + | + | - | + | + | + | + | + | 1\|Cave | NB2 (log) | ~1 | 6164.5 | 476.7 |
| 50 | - | - | + | + | + | + | + | + | + | 1\|Cave | NB2 (log) | ~0 | 6165.6 | 477.8 |
| 51 | - | + | - | + | + | + | + | + | + | 1\|Cave | NB2 (log) | ~0 | 6167.1 | 479.3 |
| 52 | - | + | + | + | + | + | + | - | + | 1\|Cave | NB2 (log) | ~0 | 6167.2 | 479.4 |
| 53 | - | - | + | + | + | + | + | + | + | 1\|Cave | NB2 (log) | ~1 | 6167.6 | 479.8 |
| 54 | - | + | - | + | + | + | + | + | + | 1\|Cave | NB2 (log) | ~1 | 6169.1 | 481.3 |
| 55 | - | + | + | + | + | + | + | - | + | 1\|Cave | NB2 (log) | ~1 | 6169.2 | 481.4 |
| 56 | - | + | + | + | + | + | + | + | - | 1\|Cave | NB1 (log) | ~Temp | 6170.2 | 482.4 |
| 57 | - | + | + | + | + | + | + | + | + | 1\|Cave | NB1 (log) | ~0 | 6170.7 | 482.9 |
| 58 | - | + | + | + | + | + | + | + | + | 1\|Cave | NB1 (log) | ~1 | 6171.4 | 483.6 |
| 59 | - | + | + | + | + | + | + | + | + | 1\|Cave | NB2 (log) | ~Temp | 6216.3 | 528.5 |
| 60 | - | + | + | + | + | + | + | + | - | 1\|Cave | NB2 (log) | ~Temp | 6231.4 | 543.6 |
| 61 | - | + | + | + | + | + | + | + | + | 1\|Cave | NB2 (log) | ~0 | 6316.4 | 628.6 |
| 62 | - | + | + | + | + | + | + | + | + | 1\|Cave | NB2 (log) | ~1 | 6318.4 | 630.6 |
| 63 | - | + | + | + | + | + | + | + | - | 1\|Cave | NB1 (log) | ~1 | 6591.9 | 904.1 |
| 64 | - | + | + | + | + | + | + | + | - | 1\|Cave | NB1 (log) | ~0 | 6623.2 | 935.4 |
| 65 | - | + | + | + | + | + | + | + | - | 1\|Cave | NB2 (log) | ~0 | 6718.8 | 1031.0 |
| 66 | - | + | + | + | + | + | + | + | - | 1\|Cave | NB2 (log) | ~1 | 6718.9 | 1031.1 |
| 67 | + | + | + | + | + | + | + | + | + | 1\|Cave, 1\|Detector | POI (log) | ~Temp | 13797.3 | 8109.5 |
| 68 | + | + | + | + | + | + | + | + | + | 1\|Cave, 1\|Detector | POI (log) | ~1 | 13974.8 | 8287.0 |
| 69 | - | + | + | + | + | + | - | + | + | 1\|Cave | POI (log) | ~Temp | 15643.0 | 9955.3 |
| 70 | + | + | + | + | + | + | + | + | + | 1\|Cave | POI (log) | ~Temp | 15643.9 | 9956.1 |
| 71 | - | + | + | + | + | + | + | - | + | 1\|Cave | POI (log) | ~Temp | 15647.3 | 9959.5 |
| 72 | - | + | + | + | - | + | + | + | + | 1\|Cave | POI (log) | ~Temp | 15656.6 | 9968.8 |
| 73 | - | + | + | + | + | - | + | + | + | 1\|Cave | POI (log) | ~Temp | 15686.8 | 9999.0 |
| 74 | - | - | + | + | + | + | + | + | + | 1\|Cave | POI (log) | ~Temp | 15705.9 | 10018.1 |
| 75 | - | + | - | + | + | + | + | + | + | 1\|Cave | POI (log) | ~Temp | 15709.3 | 10021.5 |
| 76 | - | + | + | + | + | + | - | + | + | 1\|Cave | POI (log) | ~1 | 15840.3 | 10152.5 |
| 77 | + | + | + | + | + | + | + | + | + | 1\|Cave | POI (log) | ~1 | 15841.5 | 10153.7 |
| 78 | - | + | + | + | + | + | + | - | + | 1\|Cave | POI (log) | ~1 | 15846.3 | 10158.5 |
| 79 | - | + | + | + | - | + | + | + | + | 1\|Cave | POI (log) | ~1 | 15854.4 | 10166.6 |
| 80 | - | + | + | - | + | + | + | + | + | 1\|Cave | POI (log) | ~Temp | 15879.3 | 10191.5 |
| 81 | - | + | + | + | + | - | + | + | + | 1\|Cave | POI (log) | ~1 | 15883.6 | 10195.9 |
| 82 | - | + | - | + | + | + | + | + | + | 1\|Cave | POI (log) | ~1 | 15892.8 | 10205.0 |
| 83 | - | - | + | + | + | + | + | + | + | 1\|Cave | POI (log) | ~1 | 15904.8 | 10217.0 |
| 84 | - | + | + | + | + | + | + | + | + | 1\|Cave | POI (log) | ~Temp | 15949.2 | 10261.4 |
| 85 | - | + | + | - | + | + | + | + | + | 1\|Cave | POI (log) | ~1 | 16088.5 | 10400.7 |
| 86 | - | + | + | + | + | + | + | + | + | 1\|Cave | POI (log) | ~1 | 16155.1 | 10467.3 |
| 87 | + | + | + | + | + | + | + | + | + | 1\|Cave, 1\|Detector | POI (log) | ~0 | 17515.8 | 11828.0 |
| 88 | - | + | + | + | + | + | + | + | - | 1\|Cave | POI (log) | ~Temp | 18614.2 | 12926.4 |
| 89 | - | + | + | + | + | + | + | + | - | 1\|Cave | POI (log) | ~1 | 18983.8 | 13296.0 |
| 90 | - | + | + | + | + | + | - | + | + | 1\|Cave | POI (log) | ~0 | 19593.0 | 13905.2 |
| 91 | + | + | + | + | + | + | + | + | + | 1\|Cave | POI (log) | ~0 | 19594.6 | 13906.8 |
| 92 | - | + | - | + | + | + | + | + | + | 1\|Cave | POI (log) | ~0 | 19598.3 | 13910.6 |
| 93 | - | + | + | + | + | + | + | - | + | 1\|Cave | POI (log) | ~0 | 19600.5 | 13912.7 |
| 94 | - | + | + | + | - | + | + | + | + | 1\|Cave | POI (log) | ~0 | 19604.6 | 13916.8 |
| 95 | - | - | + | + | + | + | + | + | + | 1\|Cave | POI (log) | ~0 | 19674.7 | 13986.9 |
| 96 | - | + | + | + | + | - | + | + | + | 1\|Cave | POI (log) | ~0 | 19694.9 | 14007.1 |
| 97 | - | + | + | - | + | + | + | + | + | 1\|Cave | POI (log) | ~0 | 20007.2 | 14319.4 |
| 98 | - | + | + | + | + | + | + | + | + | 1\|Cave | POI (log) | ~0 | 20090.5 | 14402.7 |
| 99 | - | + | + | + | + | + | + | + | - | 1\|Cave | POI (log) | ~0 | 28577.5 | 22889.7 |

**References**

1 Britzke, E. R., Duchamp, J. E., Murray, K. L., Swihart, R. K. & Robbins, L. W. Acoustic identification of bats in the Eastern United States: a comparison of parametric and nonparametric methods. *J. Wildl. Manage.* **75**, 660-667, doi:10.1002/jwmg.68 (2011).

2 Klüg-Baerwald, B. J., Gower, L. E., Lausen, C. L. & Brigham, R. M. Environmental correlates and energetics of winter flight by bats in southern Alberta, Canada. *Canadian Journal of Zoology* **94**, 829-836, doi:10.1139/cjz-2016-0055 (2016).

3 Clement, M. J., Murray, K. L., Solick, D. I. & Gruver, J. C. The effect of call libraries and acoustic filters on the identification of bat echolocation. *Ecology and Evolution* **4**, 3482-3493, doi:10.1002/ece3.1201 (2014).

4 O'Farrell, M. J., Miller, B. W. & Gannon, W. L. Qualitative identification of free-flying bats using the Anabat detector. *J. Mammal.* **80**, 11-23, doi:10.2307/1383203 (1999).

5 Schwab, N. A. & Mabee, T. J. Winter acoustic activity of bats in Montana. *Northwestern Naturalist* **95**, 13-27 (2014).

6 Broders, H. G., Findlay, C. S. & Zheng, L. Effects of clutter on echolocation call structure of *Myotis septentrionalis* and *M. lucifugus*. *J. Mammal.* **85**, 273-281 (2004).

7 Skalak, S. L., Sherwin, R. E. & Brigham, R. M. Sampling period, size and duration influence measures of bat species richness from acoustic surveys. *Methods Ecol. Evol.* **3**, 490-502, doi:10.1111/j.2041-210X.2011.00177.x (2012).

8 López-Baucells, A. *et al.* Stronger together: Combining automated classifiers with manual post-validation optimizes the workload vs reliability trade-off of species identification in bat acoustic surveys. *Ecological informatics* **49**, 45-53 (2019).

9 Whiting, J. C., Doering, B. & Pennock, D. Acoustic surveys for local, free-flying bats in zoos: an engaging approach for bat education and conservation. *Journal of Bat Research and Conservation* **12**, 94-99, doi:<https://doi.org/10.14709/BarbJ.12.1.2019.12> (2019).
